# Hypometabolism during Daily Torpor in Mice is Dominated by Reduction in the Sensitivity of the Thermoregulatory System

**DOI:** 10.1038/srep37011

**Published:** 2016-11-15

**Authors:** Genshiro A. Sunagawa, Masayo Takahashi

**Affiliations:** 1 Laboratory for Retinal Regeneration, RIKEN Center for Developmental Biology, 2-2-3 Minatojimaminami-machi, Chuo-ku, Kobe, Hyogo 650-0047, Japan

## Abstract

Some mammals enter a hypometabolic state either daily torpor (minutes to hours in length) or hibernation (days to weeks), when reducing metabolism would benefit survival. Hibernators demonstrate deep torpor by reducing both the sensitivity (*H*) and the theoretical set-point temperature (*T*_*R*_) of the thermogenesis system, resulting in extreme hypothermia close to ambient temperature. However, these properties during daily torpor remain poorly understood due to the very short steady state of the hypometabolism and the large variation among species and individuals. To overcome these difficulties in observing and evaluating daily torpor, we developed a novel torpor-detection algorithm based on Bayesian estimation of the basal metabolism of individual mice. Applying this robust method, we evaluated fasting induced torpor in various ambient temperatures (*T*_*A*_s) and found that *H* decreased 91.5% during daily torpor while *T*_*R*_ only decreased 3.79 °C in mice. These results indicate that thermogenesis during daily torpor shares a common property of sensitivity reduction with hibernation while it is distinct from hibernation by not lowering *T*_*R*_. Moreover, our findings support that mice are suitable model animals to investigate the regulation of the heat production during active hypometabolism, thus suggesting further study of mice may provide clues to regulating hypometabolism in mammals.

Mammals and birds evolutionarily gained homeothermicity (a constant *T*_*B*_) to keep the animal’s internal environment stable despite changes in the outer environment. The ability to maintain a stable environment inside the body allowed species the freedom to spread widely over various regions with differing environments. While providing the freedom to prosper, homeothermicity entails a huge metabolic cost. Small animals, which have a high temperature conductance compared to larger animals, spend a larger portion of their metabolism on thermogenesis^1^ and must produce more heat relative to their size to keep a constant *T*_*B*_. This is thought to be the reason why active hypometabolism is seen mainly in relatively small animals^2^. Based on the duration of the torpid status, active hypometabolism is called daily torpor or hibernation (deep torpor), which are daily base or seasonal base, respectively. Any kind of active hypometabolism reduces the demand for oxygen by giving up various vital functions and the animal becomes immobile and unresponsive. Some small animals evolved to take these risks rather than traveling aggressively to find food. Although active hypometabolism might appear to represent an evolutionary regression, it is a functional adaptation for overcoming situations that have become critical due to the high oxygen demands of homeothermicity. Four conditions are required for a euthermic animal to undergo hypometabolism, and the mechanisms of none of them are clearly known. First, the animal must turn off or at least suppress the thermoregulatory system during active hypometabolism. During hypometabolism, the animal reaches a hypothermic state that would trigger thermogenesis under normal conditions. If the normal thermoregulatory mechanism is not modified, additional energy will be spent rather than saved—which is clearly undesirable during hypometabolism. Second, the animal must be able to endure low respiration levels or low oxygen supply. Euthermic animals are designed to maintain life with a certain oxygen supply; however, in some small animals, the oxygen consumption during hibernation is reduced to 2–3% of that in an aroused state^3^. The animals have to maintain their vital metabolism under scarce oxygen conditions. Third, the animal must be resistant to hypothermia. Mammals maintain a constant *T*_*B*_. However, hibernators typically have a minimum *T*_*B*_ of 2–10 °C^4^. Non-hibernator mammalian tissues are usually damaged under such hypothermic state by disturbances in cellular ion homeostasis triggered by alterations in membrane fluidity^5^. Moreover, rewarming from hypothermia is also known to induce cellular stress response^6^. These hypothermia related responses must be prevented during and after hypometabolism. Fourth, the animal must be able to return to a normal metabolic state by producing heat from a hypometabolic state, and to tolerate this rise in heat. Animals in daily torpor or hibernation return to a euthermic state over a period of several hours^7^. This is striking in two points: 1) warming begins with the animal in a hypometabolic state in which it cannot produce heat as usual, and 2) live cells cannot usually survive such a rapid thermal change.

Among the four conditions of hypometabolism, the modification of the thermoregulatory system during active hypometabolism have been the centre of interest for a long time^8^,^9^,^10^,^11^,^12^,^13^. One issue that has been discussed in this field is the determination of the main effector of hypometabolism. What components of the thermoregulatory system are involved in hypometabolism? There are three possibilities; the heat conductance of the body (*G*), the reference of the body temperature (*T*_*R*_) and the open-loop gain of the thermoregulatory feedback system (*H*). *G* is the major factor responsible for how easy the heat is lost. It is largely determined by skin blood flow and body surface area. *T*_*R*_ is a reference temperature or the theoretical set-point of body temperature, which the system is targeting to. Although, the word ‘set-point’ is less frequently used due to the discovery of multiple mechanisms governing the body temperature in a multiple target temperatures^14^,^15^, in this article we use the term ‘set-point’ to express the temperature that the thermoregulation system is driving toward to by combining various mechanisms to keep the core body temperature stable. *H* is another theoretical parameter describing the open-loop gain of the thermoregulatory negative feedback system. The gain of the negative feedback determines the degree of effectiveness with which a control system maintains constant conditions^16^. In this system, when the *H* is larger, *T*_*B*_ tends to become closer to *T*_*R*_. Considering these components, when the animal is not moving, in other words, when the animals’ external work is negligible, a block diagram for thermoregulation can be written as Fig. 1a. Several groups have investigated the dynamics of these parameters in hibernators and reported that reduction is seen in both *T*_*R*_ and *H*^8^,^9^,^17^. The basic approach in hibernators is to observe various metabolic states during deep torpor while controlling *T*_*A*_ and to estimate the parameters. Usually, the stable torpid period in hibernators last for days which allow testing multiple conditions during a single torpor bout. On the other hand, these parameters in daily torpor are not known. This is because in daily torpor, the period of stable hypometabolism, which is the period when the *VO*_*2*_ and *T*_*B*_ are stable, last only for minutes to hours. This is preventing to perform multiple observations of metabolism at various *T*_*A*_s, which is not difficult in hibernators because they have days of stable torpid periods. To reveal the degree to which the three factors contribute to hypometabolism in daily torpor, we chose to investigate the house mouse, *Mus musculus*, which is not only one of the most popular laboratory animals, but it is also capable of entering daily torpor^18^,^19^. To overcome the metabolism recording difficulties in torpid animals, in this study, we first built a system to simultaneously record the oxygen consumption and body temperature of a freely-moving mouse. To evaluate metabolism quantitatively and objectively during daily torpor, we developed a fully automated hypometabolism-detection algorithm based on Bayesian inference. Observing the normal and torpid mouse in various *T*_*A*_s, we estimated the parameters of the thermoregulatory system to elucidate the factors determining hypothermia during daily torpor.

## Results

### System for recording the metabolism of free-moving mice under controlled ambient temperature

**Figure 1:**
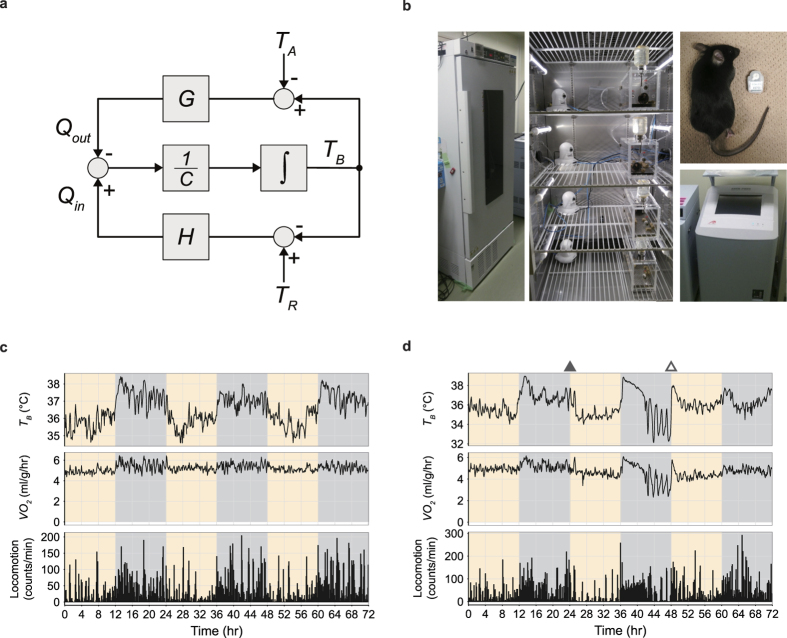
System for recording the metabolism of free-moving mice under controlled ambient temperature. (**a**) A block diagram of the thermoregulatory system in mammals when the animal is not moving, therefore assumed to exert no external work. The upper loop and the lower loop represent the heat loss and heat production loop, respectively. The time derivative of body temperature (*T*_*B*_) is derived from the difference of heat production (*Q*_*in*_) and heat loss (*Q*_*out*_) divided by the thermal capacity (*C*). *Q*_*out*_ is derived from the difference of ambient temperature (*T*_*A*_) and *T*_*B*_ multiplied by heat conductance (*G*). *Q*_*in*_ is derived from the difference of theoretically defined set-point temperature (*T*_*R*_) and *T*_*B*_multiplied by *H*, which is the open-loop gain of the thermoregulatory feedback system. (**b**) A system for evaluating the metabolism of free-moving mice. The temperature-controlled animal chamber (left panel) and the inside of the chamber (middle panel), in which four mice can be recorded at once, are shown. Each animal had an intraperitoneally implanted body-temperature transmitter (right panel, top). Each animal was housed in a metabolic chamber and the *VO*_*2*_ were recorded by gas mass spectrometry. (**c**) A representative recording of mouse metabolism for three consecutive days. The animal was placed in the chamber, and the *T*_*A*_ was maintained at 16 °C. Once the mouse was placed in the metabolic chamber, there was no physical contact with researchers during the recording period. Note the clear circadian rhythm seen in the *T*_*B*_, *VO*_*2*_ and locomotion. Yellow shading shows the light-on period. (**d**) A representative recording of metabolism during fasting-induced daily torpor. The mouse was placed in the chamber for three days; food was removed on the second day (filled triangle). The *T*_*A*_ was maintained at 16 °C. Daily torpor started during the latter half of the second day. The mouse returned to a euthermic state immediately after the food was returned to the chamber (unfilled triangle). Yellow shading shows the light-on period.

Many studies have reported that lowering the *T*_*A*_ and restricting food can induce daily torpor in mice^10^,^20^,^21^,^22^,^23^. However, most of these studies used a wired temperature-recording system and a small metabolism chamber to evaluate the animal, which may alter the animal’s phenotype, especially for small animals. Furthermore, the sex, age, and strain of the model animal differed among studies, making them difficult to compare. Therefore, to understand the mechanism of daily torpor in mice, we first sought to develop an efficient, minimally invasive system to evaluate their metabolism. We prepared a chamber in which the *T*_*A*_ could be controlled (Fig. 1b); the standard deviation of the difference between *T*_*A*_ and the target temperature was less than 1 °C (Supplementary Fig. 1a). We set up a long-term metabolism analyser and a wireless *T*_*B*_-monitoring system (Fig. 1b) in the thermo-controlled chamber, and successfully recorded the animals’ *VO*_*2*_ and *T*_*B*_ continuously for more than three days without any physical contact with the animal (Fig. 1c). We used networked cameras to closely monitor each animal’s appearance and health status. To determine a stable and efficient condition for inducing daily torpor, we used the inbred laboratory strain C57BL/6J, which can enter torpor under certain conditions^18^. To reduce phenotype variance as much as possible, we only used male mice between 7 and 9 weeks of age. When water remained freely available but food was removed for 24 hours under a constant *T*_*A*_ (Fig. 1d; the filled and open triangles at the top denote food removal and return, respectively), a typical torpid episode began on the latter half of the same day (Fig. 1d, Supplementary Fig. 1b,c and Supplementary Movies 1, 2 and 3). Because past studies have used various definitions of daily torpor, we sought to define daily torpor based on statistical prediction of the individual metabolism of the animal.

### Modelling and predicting metabolism from a single day recording

**Figure 2:**
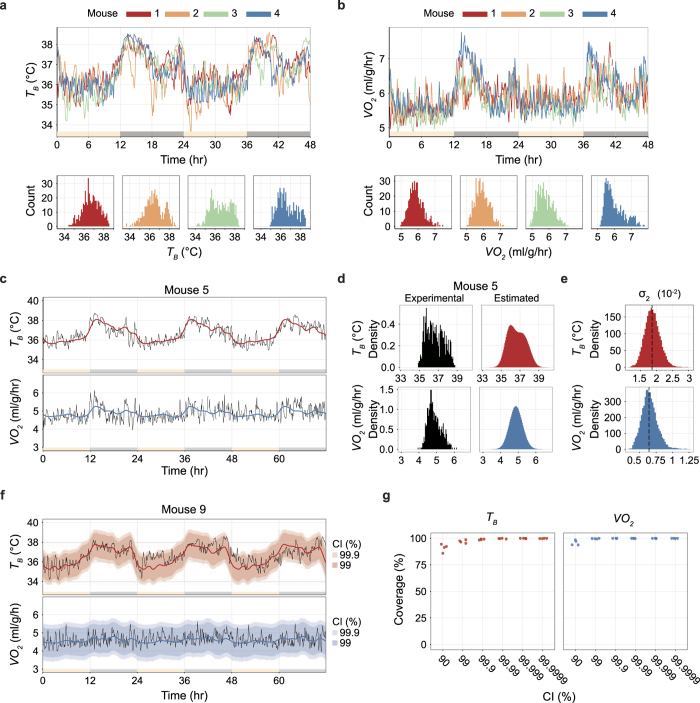
Modelling and predicting metabolism from a single day recording. (**a,b**) The distribution of the *T*_*B*_ (**a**) and *VO*_*2*_ (**b**) of four animals (mice 1 to 4) kept at a *T*_*A*_ of 12 °C for two days. The upper panels show the time series; light and dark periods are indicated by yellow and grey bars along the horizontal axis. The distribution for each animal is shown in the lower panels; each colour represents a different animal. (**c**) The estimated baseline metabolism dynamics of mouse 5. The mouse was kept at a *T*_*A*_ of 16 °C for three days. The baseline dynamics for 24 hours were fitted from the three-day-length data, and the standard deviation of the error (*σ*_*2*_) for both *T*_*B*_ and *VO*_*2*_ was estimated. The red and blue lines denote the median of the posterior distribution of the estimated *T*_*B*_ and *VO*_*2*,_ respectively. The data for the remaining three animals (mice 6 to 8) are available in Supplementary Fig. 2a. (**d**) The probability density of the experimentally obtained and estimated data for *T*_*B*_ and *VO*_*2*_ for mouse 5. Black histograms represent experimental data; red and blue histograms show the estimated probability density. The data for mice 6 to 8 are available in Supplementary Fig. 2b. (**e**) The distribution of the estimated *σ*_*2*_, which is the standard deviation of the error of the secondary trend, for *T*_*B*_ and *VO*_*2*_. (**f**) The estimated baseline metabolism dynamics of mouse 9 with credible intervals (CIs). The *T*_*A*_ was kept at 16 °C for three days. The first 24 hours were used for estimation. The red and blue lines denote the median of the posterior distribution of the estimated *T*_*B*_ and *VO*_*2*_. The red and blue shaded areas denote the CI. The data for mice 10 to12 are available in Supplementary Fig. 2c. (**g**) CI coverage rate of the metabolism on the second and third days when applying different CI ratios. The estimation was based on the first day. For both *T*_*B*_ and *VO*_*2*_, 99.9% of the CIs covered more than 99% of the sampling points of the latter two days.

Past reports have defined daily torpor by a threshold *T*_*B*_ or *VO*_*2*_^10^,^22^ or a minimal *T*_*B*_ or *VO*_*2*_^23^, but these thresholds vary from study to study. Furthermore, the dynamics of *T*_*B*_ and *VO*_*2*_ vary greatly between individuals (Fig. 2a,b). To overcome the metabolic variance observed between different individuals and environments, we propose a statistical approach to detect the individual baseline metabolism and to define daily torpor as an outlier of the baseline metabolism.

Hence, we modelled the time-series dynamics of both *T*_*B*_ and *VO*_*2*_ with a second-order trend model using Bayesian estimation (Fig. 2c,d and Supplementary Fig. 2a,b; see Methods). We used data acquired from non-torpid mice (n = 4, *T*_*A*_ = 16 °C, mice 5 to 8) for three days, and estimated *σ*_*2*_, the standard deviation of the secondary trend, to be 0.01877 and 0.00650 for *T*_*B*_ and *VO*_*2*_, respectively (Fig. 2e). By fixing *σ*_*2*_ to these values, our model can estimate the animal’s individual baseline *T*_*B*_ and *VO*_*2*_ dynamics from data taken over a single day rather than from multiple periods. This method effectively shortens the baseline recording period and lowers the stress on the animal. To verify the quality of the model, we estimated the baseline metabolism dynamics in another set of animals (n = 4, *T*_*A*_ = 16 °C, mice 9 to 12) by applying these *σ*_*2*_ values (Fig. 2f and Supplementary Fig. 2c). More than 99% of the sampling points on the second and third day were included in the 99.9% credible interval (CI) of both the estimated *T*_*B*_ and *VO*_*2*_ (Fig. 2g). Therefore, for each subject, the first day’s recording was sufficient to predict the dynamics of *T*_*B*_ and *VO*_*2*_ for the following two days. In the following analysis, a 99.9% CI was applied for estimating the baseline distribution for *T*_*B*_ and *VO*_*2*_, respectively. Development of this simple but individualized metabolism modelling allowed us to define daily torpor as an outlier from the individually predicted baseline dynamics of metabolism.

### Defining daily torpor as an outlying low metabolism

**Figure 3:**
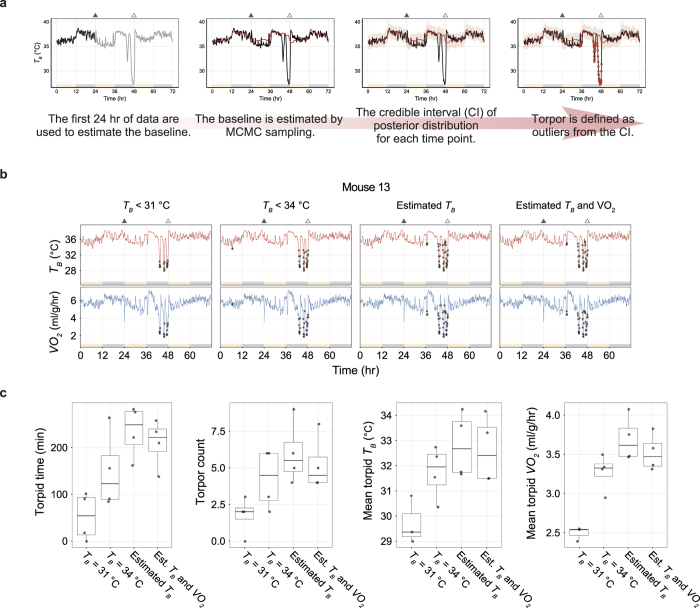
Defining daily torpor as an outlying low metabolism. (**a**) The daily torpor-detection pipeline. The first 24-hour data set is used to estimate the baseline metabolism of the individual animal (first panel). The estimated baseline is then applied to the rest of the recordings (second panel). The baseline estimation provides the CI for the prediction from the distribution of the posterior estimates (third panel). Torpor, which is defined as a lower outlier from the CI, is marked in red dots (fourth panel). The filled and unfilled triangles denote food removal and return, respectively. (**b**) Multiple torpor definitions were compared in four mice (mice 13 to 16). The animals were placed in a constant *T*_*A*_ of 12 °C for three days, and food was restricted during the second day. Results are shown for mouse 13. The two leftmost panels show daily torpor defined by a fixed threshold *T*_*B*_ of 31 °C or 34 °C. The third panel shows daily torpor defined by a lower outlier of the 99.9% CI of the estimated *T*_*B*_. The fourth panel includes the *T*_*B*_-based definition further narrowed down by adding the condition of lower outliers from the 99.9% CI of the estimated *VO*_*2*_. The filled and unfilled triangles denote food removal and return, respectively. The data for the remaining three animals are available in Supplementary Fig. 3a. (**c**) Boxplots for various torpor statistics according to the different torpor definitions listed in Fig. 3b. The band inside the box, the bottom of the box, and the top of the box represent the median, the first quartile, and the third quartile, respectively. The end of the upper whisker is the highest value that is within 1.5 times the inter-quartile range (IQR). The end of the lower whisker is the lowest value that is within 1.5 times the IQR. All data points are shown as grey dots.

Because daily torpor is a state in which animals show abnormally low metabolism, it is reasonable to define daily torpor as an outlier from the baseline metabolism. Using the statistically estimated baseline metabolism, we defined daily torpor when both *T*_*B*_ and *VO*_*2*_ were lower than the 99.9% CI of the prediction (Fig. 3a). To evaluate the functionality of this new approach, we conducted another experiments in four mice (n = 4, *T*_*A*_ = 12 °C, mice 13 to 16). We found that conventional methods^10^,^22^ detected lower rates of torpor than our method (Fig. 3b,c and Supplementary Fig. 3a). Our novel approach allowed us to objectively quantify the length and depth of daily torpor by normalizing the variance among individual recordings. Although there was little discrepancy between torpor definitions using only *T*_*B*_ or *T*_*B*_ and *VO*_*2*_ (Fig. 3c), torpor defined from *VO*_*2*_ alone detected a higher rate of torpid status in the current study (Supplementary Fig. 3b). Applying this simple but robust torpor-detection method, we next investigated how *T*_*A*_ influences metabolism during daily torpor. The comprehensive analysis of *T*_*A*_ dependent phenotype of daily torpor allowed us to estimate the fundamental factors of thermoregulatory system during torpor.

### Body-temperature homeostasis is actively controlled during torpor

**Figure 4:**
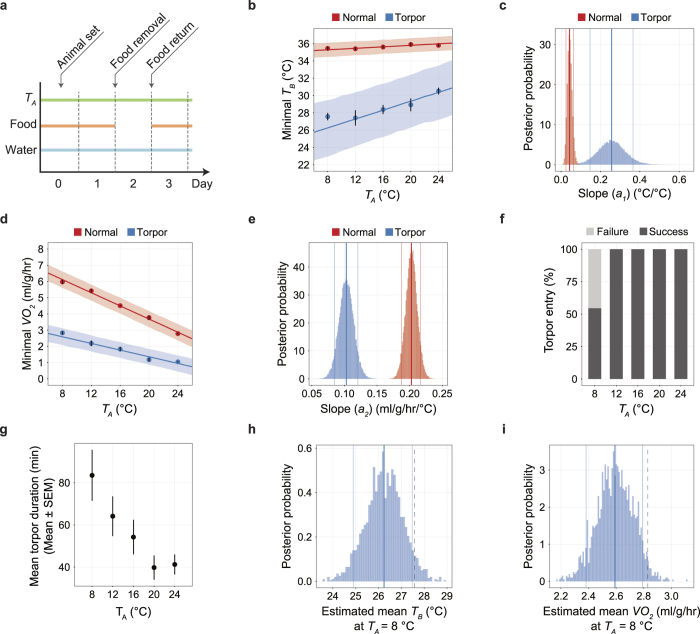
Body-temperature homeostasis is actively controlled during daily torpor. (**a**) Protocol of the fasting-induced daily torpor experiment. Animals were placed in the *T*_*A*_-constant chamber on Day 0; data were recorded for 72 hours from the beginning of Day 1. Food was removed and returned at the beginning of Day 2 and Day 3. Water was freely accessible throughout the experiment. (**b**) The minimum *T*_*B*_ at various *T*_*A*_s. Including the following panels, red and blue denote normal and torpid status, respectively. For normal status, the minimum *T*_*B*_ of the dark phase of Day 1 was used for analysis. For torpid status, the minimum *T*_*B*_ during torpor was used for analysis. As in the following panel d, the dots with the vertical error bars denote the observed mean and SEM of the minimum variables (*T*_*B*_ in **b**, *VO*_*2*_ in **d**) at each *T*_*A*_, and the line and the shaded area denote the mean and the 89% HPDI intervals of the estimated minimum variables. (**c**) The posterior distribution of the slope (*a*_*1*_) of *T*_*A*_−*T*_*B*_ relationship. Including the following distribution panels in this figure, the bold and thin lines denote the mean and the 89% HPDI intervals of the estimated values. The bin size is 0.005. (**d**) The minimum *VO*_*2*_ at various *T*_*A*_s. Minimum *VO*_*2*_ was defined as the *VO*_*2*_ recorded when the *T*_*B*_ was minimum. (**e**) The posterior distribution of the slope (*a*_*2*_) of *T*_*A*_-*VO*_*2*_ relationship. The bin size is 0.001 ml/g/hr/°C. (**f**) The rate of successful daily torpor induction at various *T*_*A*_s. When *T*_*A*_ was above 12 °C, all animals entered daily torpor. (**g**) The averaged torpor duration for each episode. One torpor episode is tended to be shorter when the *T*_*A*_ gets higher. (**h, i**) The posterior distribution of the estimated mean (n = 6) of *T*_*B*_ (**h**) and *VO*_*2*_ (**i**) at a *T*_*A*_ of 8 °C. Because the observed mean (dashed lines) is larger than the 89% HPDI in both *T*_*B*_ and *VO*_*2*_, when *T*_*A*_ = 8 °C, the animal exhibited higher metabolism than expected. The bin size is 0.1 °C and 0.01 ml/g/hr for *T*_*B*_ and *VO*_*2*_, respectively.

A constant body temperature is a key characteristic of mammals. The thermoregulatory system is apparently altered during daily torpor, because the *T*_*B*_ is lower than in normal states. To test the influence of *T*_*A*_ on *T*_*B*_ during daily torpor, we kept the animal under a constant *T*_*A*_ of 8, 12, 16, 20, or 24 °C and removed food for 24 hours to induce daily torpor (Fig. 4a and Supplementary Fig. 4a). The minimal metabolic point was defined at a point where the *T*_*B*_ is minimized within the time-range of interest.

To model the temperature dependent metabolism, first, we linearly regressed minimal *VO*_*2*_ and *T*_*B*_ with *T*_*A*_ during the second-half of Day 1 as follows:


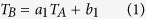



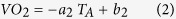


According to the estimation, the minimum *T*_*B*_ remained nearly constant against changes in *T*_*A*_, as the 89% highest posterior density interval (HPDI) of the slope *a*_*1*_ was between 0.024 and 0.063 and the mean was 0.043 (Fig. 4b,c, red lines) while the minimal *VO*_*2*_ during non-torpid status was negatively correlated with the *T*_*A*_ and the 89% HPDI of the *a*_*2*_ was between 0.188 and 0.216 ml/g/hr/°C and the mean was 0.203 ml/g/hr/°C (Fig. 4d,e, red lines).

We next analyzed the torpor-inducing efficiency of removing food for 24 hours. When the *T*_*A*_ was 12 °C or higher, 100% of the animals tested entered daily torpor, while when the *T*_*A*_ was lowered to 8 °C, only 54.5% of the animals succeeded to enter torpor (Fig. 4f). Although the *T*_*A*_ did not affect the frequency of animals entering torpor during a fasting episode (Supplementary Fig. 4b), the total duration of daily torpor tended to prolong with lower *T*_*A*_ (Supplementary Fig. 4c). Interestingly, this tendency was held even in comparing the average duration of a single period of daily torpor (Fig. 4g).

Moreover, we evaluated the metabolism of the animal during daily torpor. When *T*_*A*_ was higher than 12 °C, *T*_*B*_ decreased dramatically (Fig. 4b, blue lines) during torpor. For example, at *T*_*A*_ = 12 °C, minimal *T*_*B*_ reduced from 35.4 ± 0.17 °C to 27.4 ± 0.88 °C during torpor (mean ± SEM, n = 8). This is completely different from the normal state, in which the *T*_*B*_ hardly changed at around 36 °C (Fig. 4b). Furthermore, the fact that *a*_*1*_ increased 5.9 times from the normal status (89% HPDI of the *a*_*1*_ was between 0.147 and 0.365, the mean was 0.257, Fig. 4c) indicated that *T*_*A*_ strongly influences *T*_*B*_ during daily torpor. *VO*_*2*_ also decreased prominently during daily torpor. At *T*_*A*_ = 12 °C, the drop of *VO*_*2*_ was 46.6 ± 4.0% (mean ± SEM, n = 8, Supplementary Fig. 4d). In contrast to *T*_*B*_, the minimal *VO*_*2*_ during torpor became less sensitive to *T*_*A*_, which is clearly shown by the decrease of *a*_*2*_ (89% HPDI was between 0.085 and 0.121 ml/g/hr/°C, the mean was 0.103 ml/g/hr/°C, Fig. 4d,e). These results indicate the thermoregulation mechanism during daily torpor was weakened. As a result, *T*_*B*_ becomes sensitive to *T*_*A*_. However, the active thermoregulation was not completely abolished. When we further lowered the *T*_*A*_ to 8 °C, the mean minimal *T*_*B*_ and *VO*_*2*_ during torpor were 27.6 °C and 2.83 ml/g/hr (n = 6), respectively. Both of these values were higher than the upper end of 89% HPDI of the estimated mean (Fig. 4h,i) suggesting that at *T*_*A*_ = 8 °C, an additional thermoregulatory mechanism has been kicked in. This was not apparent when *T*_*A*_ was at the range of 12 to 24 °C.

Finally, to evaluate the extent of active reduction of metabolism during daily torpor, we calculated the Q_10_ temperature coefficient of *VO*_*2*_ with *T*_*B*_ during torpor. Suprisingly, Q_10_ was as high as 6.39 at *T*_*A*_ = 24 °C, decreased along with the lowered *T*_*A*_ and reached to 3.12 at *T*_*A*_ = 12 °C. Because Q_10_ was greater than 3, the reduction of *VO*_*2*_ cannot be explained solely by the effect of hypothermia, implicating that the metabolism was actively decreased resulting in hypothermia.

Overall, these results demonstrate that the metabolic regulation for homeostatic control of the *T*_*B*_ is weakened during daily torpor, and *T*_*B*_ changes nearly passively with *T*_*A*_. The thermoregulatory system is further altered when the *T*_*A*_ drops to 8 °C, implying the existence of a secondary mechanism that remains active to keep the metabolism at a certain level even during daily torpor, reminiscent of the hibernators. To estimate the target components of thermoregulation during daily torpor, we next fitted the data to a mathematical model to estimate the parameters of the thermoregulatory system. This analysis revealed a difference between daily torpor and hibernation thermoregulation.

### The sensitivity of the heat production system is largely reduced during daily torpor while the reduction of set-point temperature is small

From the experimental results in our study, we found that the thermoregulatory system during daily torpor has dynamic properties over the *T*_*A*_ (Fig. 4b–d). To elucidate the main effectors of this dynamic system, we have fitted the results to the thermoregulation model consisting of a heat production and heat loss loop (Fig. 1a).

This model describes the thermoregulatory feedback system of the animal when the animal is not moving. The energy lost from the animal (*Q*_*out*_) is thermodynamically determined by the difference of *T*_*B*_ from *T*_*A*_ and the heat conductance (*G*) of the animal. The metabolic rate (*Q*_*in*_) is the energy used for heat production per unit time and it is designed to be a function of the difference of *T*_*R*_ from *T*_*B*_. In this study, for simplicity, we expressed both *Q*_*in*_ and *Q*_*out*_ as oxygen consumption rates (O_2_ ml/g/hr) and assumed the measured *VO*_*2*_ is equals *Q*_*in*_. This is possible when the energy production is proportional to *VO*_*2*_. The net change in heat, i.e. *Q*_*in*_ − *Q*_*out*_, divided by heat capacity *C* yields the time derivative of *T*_*B*_ as:


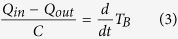


In the heat loss loop (the upper loop in Fig. 1a), if the animal is not moving, because no energy is used for external work by exercise, the *Q*_*out*_ from the animal is mainly governed by the difference of *T*_*B*_ and *T*_*A*_ as:


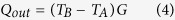


Furthermore, if *T*_*B*_ is in the steady-state (i.e., *dT*_*B*_*/dt* = 0), *Q*_*out*_ equals *Q*_*in*_ from equation (3). Substituting *Q*_*out*_ in equation (4) with *VO*_*2*_ yields:


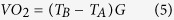


This means all of the oxygen consumption is utilized for heat production to fill the gap between the body and ambient temperature.

On the other hand, in the heat production loop (the lower loop in Fig. 1a), if the animal is still, *Q*_*in*_ is proportional to the difference between the set-point temperature *T*_*R*_ and *T*_*B*_ as:


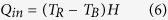


Under the steady-state condition, equation (6) can be rewritten as:


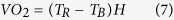


Eliminating *VO*_*2*_ by joining equations (5) and (7), under the steady-state condition, *T*_*B*_ can be described as:


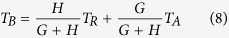


In this model, three parameters are modifiable by the body. One is the heat conductance (*G*), which is the main parameter quantifying the heat loss of the animal, and the other two are the body temperature set-point (*T*_*R*_) and the gain of the negative feedback loop of heat production (*H*). Because not all animals enter torpor when *T*_*A*_ = 8 °C (Fig. 4f), we employed *T*_*B*_ and *VO*_*2*_ recorded under *T*_*A*_ = 12, 16, 20 and 24 °C for the parameter estimation.

**Figure 5:**
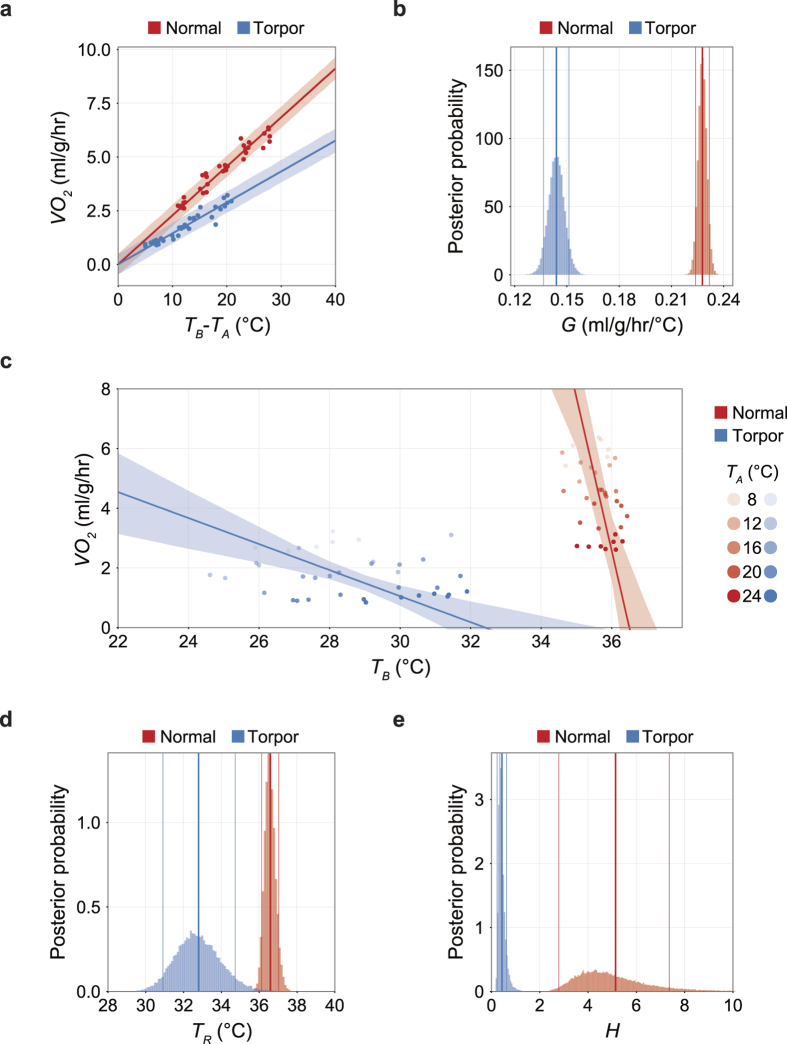
The sensitivity of the heat production system is largely reduced during daily torpor while the reduction of set-point temperature was small. (**a**) The relationship between the difference of *T*_*B*_ from *T*_*A*_ and *VO*_*2*_. Including the following panels, red and blue denote normal and torpid status, respectively. The slope of this relationship is the heat conductance, *G*. As in panel **c**, the dots represent the observed values, and the lines and shaded areas represent the means and the 89% HPDI intervals of the estimated values. (**b**) The posterior distribution of the estimated *G*. During torpor, *G* is smaller than during normal states. Decrease in *G* results in heat preservation. However, the *T*_*B*_ decrease seen in daily torpor is indicating the decrease in *G* is overridden or induced by decrease of heat production. The bin size is 0.001 ml/g/hr/°C. As in panel d and e, the bold and thin lines denote the mean and the 89% HPDI intervals of the estimated values. (**c**) The relationship between minimum *T*_*B*_ and *VO*_*2*_ seen during normal and torpid states among various *T*_*A*_s. The brightness of the dots is indicating the *T*_*A*_. The horizontal intercept of the line indicates the theoretical set-point of *T*_*B*_, which is *T*_*R*_ (See Fig. 1a). During normal states, *T*_*B*_ is kept relatively constant by employing oxygen and producing heat to fill the gap between *T*_*R*_ and *T*_*A*_. On the other hand, during daily torpor, the sensitivity against *T*_*R*_ *- T*_*B*_ is weakened which is visualized by less steep slope, which is *H*, the open-loop negative feedback gain of the heat production loop (See Fig. 1a). (**d**) The posterior distribution of the estimated *T*_*R*_. During daily torpor, *T*_*R*_ became smaller than normal states, although the mean difference was 3.79 °C. The bin size is 0.1 °C. (**e**) The posterior distribution of the estimated *H*. During daily torpor, *H* became dramatically smaller than normal states, which the mean difference reached to 4.70 ml/g/hr/°C. This is clearly showing that the open-loop gain of the heat production system reduced to 8.5% during daily torpor from the normal state. The bin size is 0.05 ml/g/hr/°C.

First, we estimated *G* from equation (5) by fitting experimental data (Fig. 5a). We found that *G* decreased from 0.228 to 0.144 ml/g/hr/°C during daily torpor (Fig. 5b). The 89% HPDI was 0.224 to 0.231 ml/g/hr/°C and 0.137 to 0.151 ml/g/hr/°C for normal and torpid status, respectively. The decrease of *G* reduces heat loss by 37% during daily torpor, thereby serves as a mechanism of heat preservation. Therefore, the decreased *G* counteracts to the reduction of *T*_*B*_ and necessitates even more profound hypometabolism to lower *T*_*B*_ during daily torpor.

Next, we estimated *T*_*R*_ and *H* from equation (7). Because we have estimated the relationship of *T*_*B*_ and *VO*_*2*_ with *T*_*A*_ from equations (1) and (2) in the previous section, we used those results to further estimate *T*_*R*_ and *H*. Eliminating *T*_*A*_ from equations (1) and (2) results in:


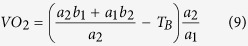


From equations (7) and (9), both *T*_*R*_ and *H* can be described by *a*_*1*_, *a*_*2*_, *b*_*1*_ and *b*_*2*_ as:


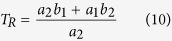



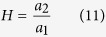


Applying the estimated *a*_*1*_, *a*_*2*_, *b*_*1*_ and *b*_*2*_ (Fig. 4c,e and Supplementary Fig. 5a,b), we obtain *T*_*R*_ and *H*. Figure 5c represents the estimated *T*_*B*_-*VO*_*2*_ relationship with the observed data. In this figure, the horizontal intercept denotes the *T*_*R*_, because theoretically the thermoregulatory system will no longer produce heat when the *T*_*B*_ equals *T*_*R*_. In normal status, the 89% HPDI of the estimated *T*_*R*_ was between 36.1 and 37.2 °C (Fig. 5d, red histogram). Interestingly, during torpor, the 89% HPDI of *T*_*R*_ was estimated to be between 30.9 and 34.7 °C (Fig. 5d, blue histogram). These are lower than in normal status but not as low as the observed *T*_*B*_ during torpor (Fig. 4b). These results clearly showed that the hypometabolism during daily torpor cannot be explained only by a low *T*_*R*_. In fact, the gain of the negative feedback loop of heat production (*H*), which is represented by the negative slopes of the lines in Fig. 4c during normal and torpid status turned out to be very contrasting. The 89% HDPI of the estimated *H* during normal status was between 2.79 and 7.36 ml/g/hr/°C (Fig. 5e, red histogram), while during torpor, it decreased to values between 0.233 and 0.624 ml/g/hr/°C (Fig. 5e, blue histogram). The averaged estimated *H* of torpid status showed 91.5% reduction from the normal status. Overall, we conclude that the striking reduction in the open-loop negative feedback gain of the heat production system plays the central role in regulating the hypometabolism during daily torpor. The contributions of a small reduction of *T*_*R*_ and *G* are secondary.

## Discussion

### Metabolism during daily torpor is actively regulated to stay above a certain level

We developed a simple but robust method to estimate the individualized baseline metabolism of mice and defined daily torpor as an outlier of the baseline metabolism (Figs 1, 2 and 3). Since the baseline is defined from the animals in *ad libitum* access to food (day 1), the abnormal low metabolism detected in our study may include the metabolic drop caused by fasting itself. This may be a problem to discriminate the fasting response and the torpid response to evaluate what extent the torpor time is influenced by fasting. However, our main aim to apply the baseline metabolism is to absorb the individual variance in metabolism. Therefore, we defined daily torpor of an animal by including the fasting response as a part of the hypometabolisc response.

We showed that during daily torpor, the metabolism is dynamically regulated in response to changes in *T*_*A*_. In torpid periods when *T*_*A*_ was 12 °C or higher, the *T*_*B*_ responded nearly passively to *T*_*A*_ (Fig. 4d) in a semi-heterothermic manner. Notably, when the *T*_*A*_ dropped to 8 °C, the animals stopped responding semi-heterothermically and the minimum *T*_*B*_ and *VO*_*2*_ were higher than they were predicted (Fig. 4h,i).

Interestingly, the minimum *T*_*B*_ reported during daily torpor varies among studies^10^,^21^,^24^,^25^. The minimum *T*_*B*_ during daily torpor strongly correlated with *T*_*A*_. Therefore it is possible that our observed minimum *T*_*B*_ was higher than in other studies because of differences in *T*_*A*_. However, since we measured the minimum *T*_*B*_ for a wide range of *T*_*A*_ (8 to 24 °C), it is unlikely that the differences in the minimum *T*_*B*_ are due to *T*_*A*_ variance. Other factors, such as genetic differences in the animals used in the study must be considered. Indeed, differences in daily torpor phenotype have been reported among inbred strains^18^. Therefore, tracking the minimum *T*_*B*_ phenotype among genetically modified or genetically distinct mice, such as genetically knockout animals or other inbred strains, may contribute to our understanding of the resistance to hypothermia, which is one of the four major requirements for active hypometabolism capability.

In addition, the phenomenon of the animal returning to a homeothermic state when the *T*_*A*_ drops below a certain level is seen not only in daily torpor, but also during hibernation^13^. This suggests that a common mechanism underlies both daily torpor and hibernation, and that studying torpor in mice may help to reveal a universal mechanism of active hypometabolism.

Finally, it is worth noting that the oscillative behavior of metabolism during torpor was always seen in our study. This is discrepant from previous studies which typically show continuous lowered levels of *VO*_*2*_ or *T*_*B*_^10^,^26^. We think the oscillatory metabolism during torpor is partially because the animals we have used are exclusively males. We decided to use male rather than female to avoid the possible reproductive cycle effect, which are often seen in sleep/wake cycles^27^,^28^. If the sex difference in torpor phenotype is globally observed through *Mus musculus*, it may be a good lead to investigate the dynamic change of the thermoregulatory parameters during daily torpor.

### The active thermoregulation during daily torpor is mainly driven by less sensitivity of the heat production system

In this study, estimating *G, T*_*R*_ and *H* from observations of *T*_*B*_ and *VO*_*2*_ among various *T*_*A*_s, we concluded that the reduction in *H* is the main effector of hypometabolism during daily torpor (Fig. 1a and 5c). Importantly, equation (8) shows the ratio of *G* and *H* determines the contribution of *T*_*R*_ and *T*_*A*_ to *T*_*B*_. When *G* > *H,* the *T*_*A*_ has stronger effect to *T*_*B*_, and when *H* > *G,* the *T*_*R*_ has stronger effect to *T*_*B*_. In this study, *G/H* increased from 0.044 to 0.330 when the animal entered torpor. This is clearly showing that during daily torpor, the thermoregulatory system shifted to accept the effect of *T*_*A*_ and as a result, the effect of *T*_*R*_ had been relatively weakened.

We observed the decrease in *G* in torpid mouse as it was reported in past literatures and in other species^12^. This will decrease the heat loss, which can be explained as the secondary effect of low *T*_*B*_ against *T*_*R*_. The reaction to decrease heat loss is also supported by the torpor specific statue observed in this study (Supplementary Movie 3) that can be the result of minimizing the surface area to reduce heat loss. The slight drop in *T*_*R*_ is indicating that its contribution to torpor thermoregulation is insignificant to the decrease in *H* (Fig. 5b,d,e). This is fundamentally different from hibernators which usually show reduction not only in *H* but also in *T*_*R*_^8^,^9^,^17^. However, the degree of reduction of *H* during daily torpor was similar to that of hibernators. This is indicating that the sensitivity reduction in thermogeneration in daily torpor may share a common mechanism with hibernation.

For example, Yellow-bellied marmots (*Marmota Zauiuentris*) have *T*_*R*_ = 36.3–37.0 °C and *H* = 0.136–0.253 ml/g/hr/°C during euthermic states^9^. During hibernation, *T*_*R*_ drops to 6.2–9.5 °C and *H* decreases to 0.006–0.023 ml/g/hr/°C which is nearly a 90% decrease. Importantly, both *T*_*R*_ and *H* are reduced. Another example is seen in golden-mantled ground squirrels (*Citellus lateralis*), which reduce *T*_*R*_ from 37.2–37.9 to 1.2–12.5 °C during hibernation^8^,^17^. In these species, *H* is reduced from 0.781–1.20 to 0.032–0.073 ml/g/hr/°C which is more than a 90% reduction.

There are, at least, three possible explanations for dominantly lowering *H* but not *T*_*R*_ during daily torpor. One is based on structure of the thermoregulatory system (Fig. 1a). It is possible to control *T*_*B*_ by changing both *T*_*R*_ and *H*, as in hibernators. However, regulating multiple actuators is much complicated than controlling a single actuators, and as a system, usually it is easier to obtain stability when the number of actuator is less. Moreover, since daily torpor is only induced for hours, where hibernators stay in hypometabolic states for days, it is reasonable to keep the regulatory system during torpor simpler and much controllable than in hibernation. The drawback of keeping the *T*_*R*_ high is the higher *T*_*B*_. As in equation (8), when having *G* and *H* at the level as low as hibernators with a fixed *T*_*A*_, *T*_*B*_ depends on *T*_*R*_. For hibernators, which have to survive for several months without eating, lowering the *T*_*R*_ along with *H* is reasonable.

The second possible reason for not lowering *T*_*R*_ during daily torpor rises from one of the requirements for a mammal to enter active hypometabolism, the resistance to hypothermia. Even during daily torpor, the animal will appreciate as low metabolism as possible if the aim of the hypometabolism is to save energy. Therefore, leaving *T*_*R*_ in a relatively high value indicate there is a minimum temperature the animal can accept. Indeed, the torpid animals showed higher metabolism than expected from the trend when *T*_*A*_ was lowered to 8 °C (Fig. 4h,i).

The third possible explanation can also be derived from another requirement for mammalian active hypometabolism, the rewarming function from hypometabolic state. Hypometabolic animals have to produce heat to return to euthermic condition. Hibernation and daily torpor exhibit different time-courses during the rewarming. In hibernators, they return to euthermic condition within hours, while in daily torpor, animals rewarm in less than an hour (Fig. 3b and Supplementary Fig. 3a). It is well documented that in hibernators, the decrease of *T*_*R*_ takes longer time than decrease of *H*^9^. The slower dynamics of *T*_*R*_ regulation imply a certain underlying mechanism to prevent the *T*_*R*_ to decrease as quick as *H*. Because *T*_*R*_ is regulated in the brain, it is natural to assume the *T*_*R*_ is controlled through the dynamic change in the neural network such as synpatic plasticity, which may require considerable energy and time in either increasing or decreasing *T*_*R*_. Therefore, in daily torpor, to implement a minute-order rewarming, changing the *T*_*R*_ would be nothing but a hurdle, which can be a possible reason why it is not lowered as in hibernators.

Our results clearly show that the reduction in the open-loop gain of the thermogeneration system, which is the sensitivity to the temperature gradient between *T*_*B*_ and *T*_*R*_, is the major effector of hypometabolism in mouse daily torpor. Throughout the thermosensory afferent and the thermogeneration efferent pathway^29^, theoretically, suppressing any of the sites can reduce the feedback gain. Basically, they can be grouped into central or peripheral mechanisms according to the suppressed site. In the central nervous system (CNS), it is possible that the preoptic area in the brain, including the thermoregulatory centre, is sending fewer signals to produce heat. If this is the case, there should be less neural activity at the thermoregulatory centre or at upstream of that. On the other hand, it is possible that even though the brain is sending signals to produce heat, the peripheral tissue (i.e. brown adipose tissue or skeletal muscle) may not be producing heat because it does not receive the signal properly or is unable to respond to the signal. Either scenario can reduce *H* without altering the set-point temperature. While alternation in thermoregulatory system is another requirement for the capability to undergo active hypometabolism, further investigation is necessary to narrow down the mechanism of reduced *H* in daily torpor.

One approach to investigate the thermoregulatory modification in the CNS is to utilize the novel imaging technology to evaluate the CNS systematically during daily torpor^30^,^31^. Determining whether specific parts of the brain, including the thermoregulatory centre, are active or inactive during daily torpor will provide important clues to how the regulatory network of hypometabolism functions. The other approach is to examine the peripheral tissue during torpor. As in the present study, the overall reduction of the animal’s metabolism can be evaluated by analysing the *VO*_*2*_ from respiration. However, the variations in hypometabolism among organs and tissues can only be evaluated by testing the metabolism in each component. Because the basic metabolic rate varies among organs and tissues during euthermia, the reduction in metabolic rate may also differ among organs and tissues during hypometabolism. Investigating the localization of hypometabolism during daily torpor will offer new insights into the regulatory mechanism of active hypometabolism and may also offer clues to the mechanism by which the animal rewarms from a very hypometabolic state, which is another requirement for an animal to safely enter a hypometabolic state.

### Toward active hypometabolism implementation in humans

Humans may benefit from active hypometabolism. In stroke, it is important to begin treatment as quickly after the onset of symptoms as possible^32^ because outcomes worsen as time passes due to hypoxia of the brain. If we could reduce the demand for oxygen by inducing active hypometabolism, the patient could buy time by slowing the progress of brain damage and thus may survive periods of hypooxygenation.

Tissue and organ preservation can also benefit from induced hypometabolism. Regenerative therapies have been approved for selected organs (Mandai *et al*., in preparation), and clinical studies are underway for others. One of the inherent problems in this field is how to preserve tissues or organs once produced, keeping them healthy and fresh. Cooling is currently a mainstay for preserving organs, but cannot keep organs alive for weeks. Rather than removing heat, reducing the metabolism by active hypometabolism may solve this problem. Torpid animals do not have low metabolism because they are cold; they are cold because they have low metabolism.

To implement active hypometabolism in humans, we need to deal with the four conditions, which were mentioned in the Introduction. Recently, several species of primates were found to be hibernators^33^,^34^. Although it is tempting to investigate hypometabolism in an animal that is close to humans, monkeys are difficult to use due to their limited availability. Non-primate hibernators are alternative candidates for understanding the mechanism of hypometabolism but the seasonal effect of the hypometabolism is a burden for aggressive research. Therefore, we think mouse is an ideal animal to investigate hypometabolism for future clinical application. Thus, this study has great importance as a pioneering and fundamental work to clarify the mechanism of active hypometabolism in mouse, and moreover, for the development of the next generation hypometabolic medicine.

## Methods

### Animal experiments

All animal experiments were performed according to the guidelines for animal experiments of RIKEN Center for Developmental Biology and approved by the Animal Experiment Committee of the RIKEN Kobe Institute (Approval ID: AH27-05-4). C57BL/6NJcl mice were purchased from CLEA Japan, Inc. and C57BL/6J mice were from Oriental Yeast Co., Ltd. Until the mice were used in torpor experiments, they were given food and water *ad libitum* and maintained in an ambient temperature of 21 °C, a relative humidity of 50%, and a 12-hr light/12-hr dark cycle.

Two C57BL/6NJcl male mice were used for the experiments shown in Fig. 1c,d; 57 C57BL/6J male mice were used for the rest of the experiments. The age at the time of the experiment was 8.01 ± 0.15 weeks (mean ± SD, n = 59).

During the experiments, each animal was housed in a temperature-controlled chamber (HC-100, Shin Factory), and the temperature inside the chamber was monitored continuously by a temperature logger (Thermochron iButton, DS1922L-F5#, Embedded Data Systems). To record *T*_*B*_ continuously, a telemetry temperature sensor (TA11TA-F10, DSI) was implanted in the animal’s abdominal cavity under general inhalation anaesthesia at least 7 days before recording. The metabolism of the animal was continuously analyzed by respiratory gas analysis (ARCO-2000 mass spectrometer, ARCO system). During the experiment, the animal was monitored through a network video camera (TS-WPTCAM, I-O DATA, Inc.) and movies were recorded as needed. This video camera’s ability to record infrared movies made it possible to monitor the animal’s health during the dark phase without opening the chamber.

### Body temperature and oxygen consumption modelling with daily torpor detection

To model the temporal variation of *T*_*B*_ and *VO*_*2*_, we constructed the models in a Bayesian framework and estimated the parameters using Markov Chain Monte Carlo (MCMC) sampling by Stan^35^ with the RStan library^36^ in R^37^. The fundamental principles and techniques for designing the model were based on the book *Statistical Rethinking*^38^.

When the unobservable baseline of *T*_*B*_ or *VO*_*2*_ is defined as a time-variable *α*_*k*_, with the noise factor *ε*_*t*_, the total time point in a day *K*, and the total number of days in the time series *D*, the observed state *Y*_*t*_ can be described as:


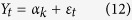



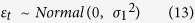



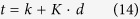



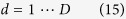



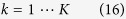


*α*_*t*_ is defined in a circulatory secondary trend model as


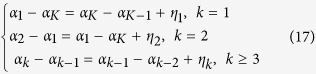



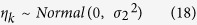



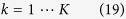


Equation (17) can be transformed as:


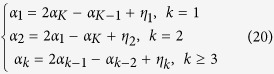


Using three-day recordings of metabolic data from four non-torpid mice, we estimated the posterior distribution of the *σ*_*2*_ of both *T*_*B*_ and *VO*_*2*_ by MCMC sampling of the equations (12) to (20) (Fig. 2c and Supplementary Fig. 2a). Uniform priors were applied for every parameter. We fixed the *σ*_*2*_ to the median of the posterior distribution (Fig. 2e), and using another four animals, we estimated *α*_*k*_ from a single-day-length recording for each animal and calculated the posterior distribution of *Y*_*t*_ (Supplementary Source Code 2). We calculated the minimum interval of *Y*_*t*_ that can predict the metabolic dynamics shown in Fig. 2f and Supplementary Fig. 2c. In the remaining analyses, we used the 99.9% CI of the posterior distribution of *Y*_*t*_ estimated from the animal’s first day of recordings to detect outliers. That is, when the value was lower than the CI, that time point was defined as torpor due to an abnormally low metabolic status (Fig. 3a). In this study, when both *T*_*B*_ and *VO*_*2*_ met the criteria, the time point was treated as torpor.

### Daily torpor induction experiment

Each daily torpor induction experiment was designed to record the animal’s metabolism for three days (Fig. 4a). The animals were introduced to the chamber the day before recording started (Day 0). Food was placed on the floor, and a water bottle was made available. The thermosensor implanted in the mouse was turned on before placing the mouse in the chamber. We began recording metabolic data at the beginning of the light phase, which was ZT-0 of Day 1. On Day 2, ZT-0, the food was removed to induce torpor. After 24 hours, on Day 3, ZT-0, the food was returned to each animal.

### Parameter estimation of the thermoregulatory system

To thermoregulatory system was modelled as an integration of the heat loss and heat production of the animal (Fig. 1a). We aimed to estimate the parameters *G, T*_*R*_ and *H* from the experimental observations. In the experiment, the controllable parameter was *T*_*A*_ and the observable parameters were *T*_*B*_ and *VO*_*2*_. Therefore, we first fitted the experimental results to linear models, equations (1, 2 and 5), and estimated *a*_*1*_, *a*_*2*_, *b*_*1*_, *b*_*2*_ and *G* by MCMC sampling. For priors, *a*_*1*_, *a*_*2*_ and *G* used log-normal distribution (natural logarithm of the variables were normally distributed by mean of 0 and standard deviation of 1) and the intercepts *b*_*1*_ and *b*_*2*_ used uniform distribution. Because *T*_*R*_ and *H* can be described as equation (7), they can also be described as equations (10) and (11). Introducing the posterior distribution of *a*_*1*_, *a*_*2*_, *b*_*1*_ and *b*_*2*_, we estimated the posterior distribution of *T*_*R*_ and *H*. See Supplementary Source Code 2 for further information.

To compare the estimated parameters with past reports, we referenced the *T*_*R*_ and *H* of hibernators from three studies^8^,^9^,^17^. We assumed 5.3 cal of energy is equivalent to consumption of 1 ml of oxygen and 1 cal = 4.184 J was applied for unit conversion.

### Calculation of Q_10_ temperature coefficient of oxygen consumption rate

The Q_10_ temperature coefficient of oxygen consumption rate among normal and torpid condition was calculated in the following equation:


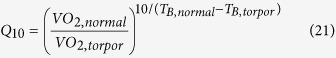


Because most biological reactions proceed with a Q_10_ of ~2 or 3^39^, if the Q_10_ during torpor is larger than this, the decrease of oxygen consumption rate cannot be explained by simple temperature effect; rather, it can be assumed that the metabolism was suppressed actively.

## Additional Information

**How to cite this article**: Sunagawa, G. A. and Takahashi, M. Hypometabolism during Daily Torpor in Mice is Dominated by Reduction in the Sensitivity of the Thermoregulatory System. *Sci. Rep.* **6**, 37011; doi: 10.1038/srep37011 (2016).

**Publisher’s note**: Springer Nature remains neutral with regard to jurisdictional claims in published maps and institutional affiliations.

## Supplementary Material

Supplementary Information

Supplementary Movie 1

Supplementary Movie 2

Supplementary Movie 3
