# Supplementary material for: Hypometabolism during Daily Torpor in Mice is Dominated by Reduction in the Sensitivity of the Thermoregulatory System
Source: Sci Rep. 2016 Nov 15;6:37011. doi: 10.1038/srep37011 (PMC5109469; doi:10.1038/srep37011)
Supplement: Supplementary Information [file srep37011-s1.pdf]

Supplementary Information

Hypometabolism during Daily Torpor in Mice is Dominated by Reduction in the Sensitivity of the Thermoregulatory System

Genshiro A Sunagawa[1,*], Masayo Takahashi[1]

[1] Laboratory for Retinal Regeneration, RIKEN Center for Developmental Biology, 2-2-3 Minatojimaminami-machi, Chuo-ku, Kobe, Hyogo, 650-0047, Japan.

* Corresponding author: genshiro.sunagawa@riken.jp

Supplementary Figure Legends

Supplementary Figure 1. System for recording the metabolism of free-moving mice under controlled ambient temperature

(**a**) A violin plot of observed T_A for different target temperatures. For each target temperature, T_A was measured every 10 min for 7 days. The standard deviation of the difference between T_A and the target temperature was 0.963, 0.441, 0.172, 0.253, and 0.363 for T_A = 8, 12, 16, 20 and 24 °C respectively. (**b, c**) Representative picture of a mouse in a non-torpid (**b**) and torpid state (**c**). During non-torpid state, mice have the ability to move around freely. The torpid animal assumes a position somewhat like a ball-shaped statue, with its eyes closed. An animal that is disturbed by physical contact cannot react as promptly during daily torpor as in a euthermic state. Also, see **Supplementary Movie 1, 2 and 3** for representative movies for an awake, asleep and torpid mouse.

Supplementary Figure 2. Modelling and predicting metabolism from a single day recording

(**a**) Estimated baseline metabolism dynamics for mice 6, 7, and 8. The mouse was kept at T_A = 16 °C for three days. The baseline dynamics for 24 hours were fitted from the three-day-length data. The estimated standard deviation of the error (σ_2) for both T_B and VO_2. The red and blue lines denote the median of the posterior distribution of the estimated T_B and VO_2, respectively. Yellow shading along the horizontal axis denotes the light-on period. (**b**) Probability density of experimentally obtained and estimated data for mice 6, 7, and 8. Upper and lower rows denote T_B and VO_2, respectively. Black histograms are the experimentally observed data, and red and blue histograms show the estimated probability density. (**c**) Estimated baseline metabolism dynamics and credible intervals (CIs) for mice 10, 11, and 12. The mouse was kept at T_A = 16 °C for three days. The first 24 hours were used for estimation. The red and blue lines denote the median of the posterior distribution of the estimated T_B and VO_2. The red and blue shaded areas denote the CIs. Yellow shading along the horizontal axis denotes the light-on period.

Supplementary Figure 3. Defining daily torpor as an outlying low metabolism

(**a**) We compared several definitions for daily torpor using data for mice 14, 15, and 16. The animals were kept at a constant T_A = 12 °C for three days; food was removed on the second day. The two leftmost panels show torpor defined by a fixed threshold for T_B (31 or 34 °C). The third panel shows torpor defined as a lower outlier from the 99.9% CI of the estimated T_B. The fourth panel includes the T_B-based definition further narrowed down by adding the condition of lower outliers from the 99.9% CI of the estimated VO_2. The filled and unfilled triangles denote food removal and return, respectively. (**b**) Ratio of the time defined as torpor according to daily torpor definitions based on outliers from baseline metabolism estimations. In the present study, time periods were defined as torpid when they matched the estimations for both T_B and VO_2.

Supplementary Figure 4. Body-temperature homeostasis is actively controlled during daily torpor

(**a**) Dynamics of metabolism during daily torpor in various T_As. For every T_A, representative data from a single animal are shown. The red and blue lines denote T_B and VO_2, respectively. The dashed lines show the borderline for torpor detection and the black dots show the time points judged as torpor. The filled and unfilled triangles denote food removal and return, respectively. (**b**) The torpor count, which is the number of torpor bouts in one fasting period, at various T_As. (**c**) The torpor duration at various T_As. The duration tends to negatively correlate to T_A. (**d**) The VO_2 reduction rate of torpid status at various T_As. When T_A was 8 °C, the VO_2 reduction reached to 50%.

Supplementary Figure 5. The sensitivity of the heat production system is largely reduced during daily torpor while the reduction of set-point temperature was small

(**a**) The posterior distribution of the vertical intercept (b_1) of T_A-T_B relationship. The bold and thin lines denote the mean and the 89% HPDI intervals of the estimated values. The bin size is 0.05. In this figure, including the following panel (**b**), red and blue denote normal and torpid status, respectively. (**b**) The posterior distribution of the vertical intercept (b_2) of T_A-VO_2 relationship. The bold and thin lines denote the mean and the 89% HPDI intervals of the estimated values. The bin size is 0.05 ml/g/hr/°C.

Supplementary Movie Legends

Supplementary Movie 1. An awake mouse

This movie shows the mouse in the active phase. The mouse is moving around. The vertical pipe hanging from the ceiling is water and the barrel shaped objects on the floor is food.

Supplementary Movie 2. A sleeping mouse

This movie shows a sleeping mouse lying in a curled-up position.

Supplementary Movie 3. A mouse in daily torpor

This movie shows a mouse in daily torpor. The food was removed nearly 24 hours before the recording. The mouse is sitting curled into a ball shape rather than lying on the floor.

Supplementary Source Code Legends

Supplementary Source Code 1

A Stan model for estimating the baseline time-series of T_B and VO_2.

Supplementary Source Code 2

A Stan model for estimating G, H and T_R for the given T_A, T_B and VO_2.

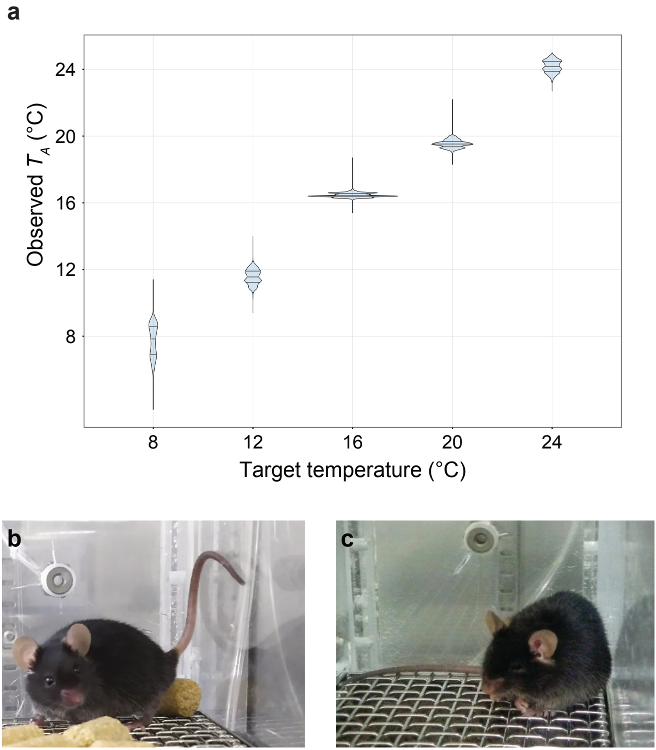

Sunagawa *et al.*, Supplementary Figure 1

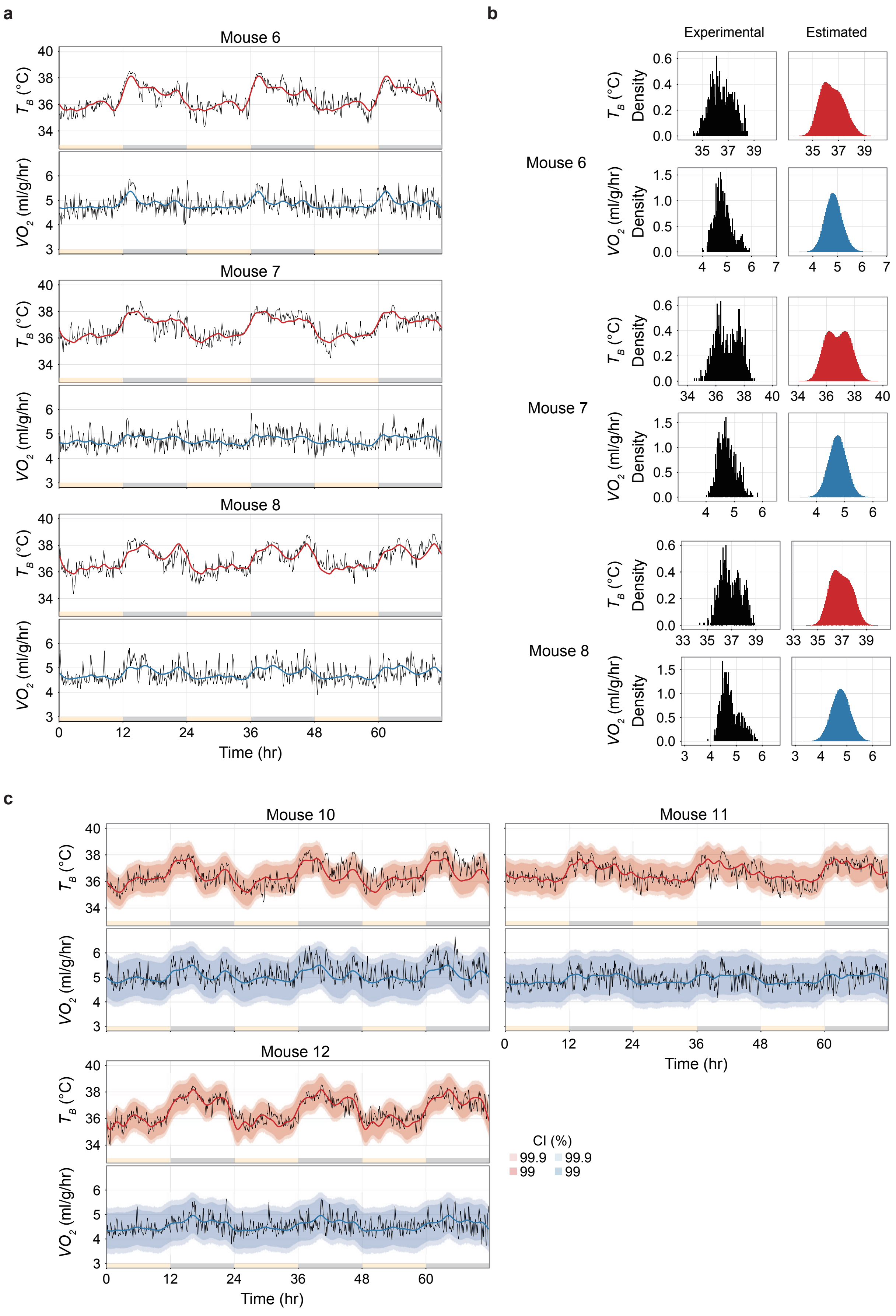

Sunagawa *et al.*, Supplementary Figure 2

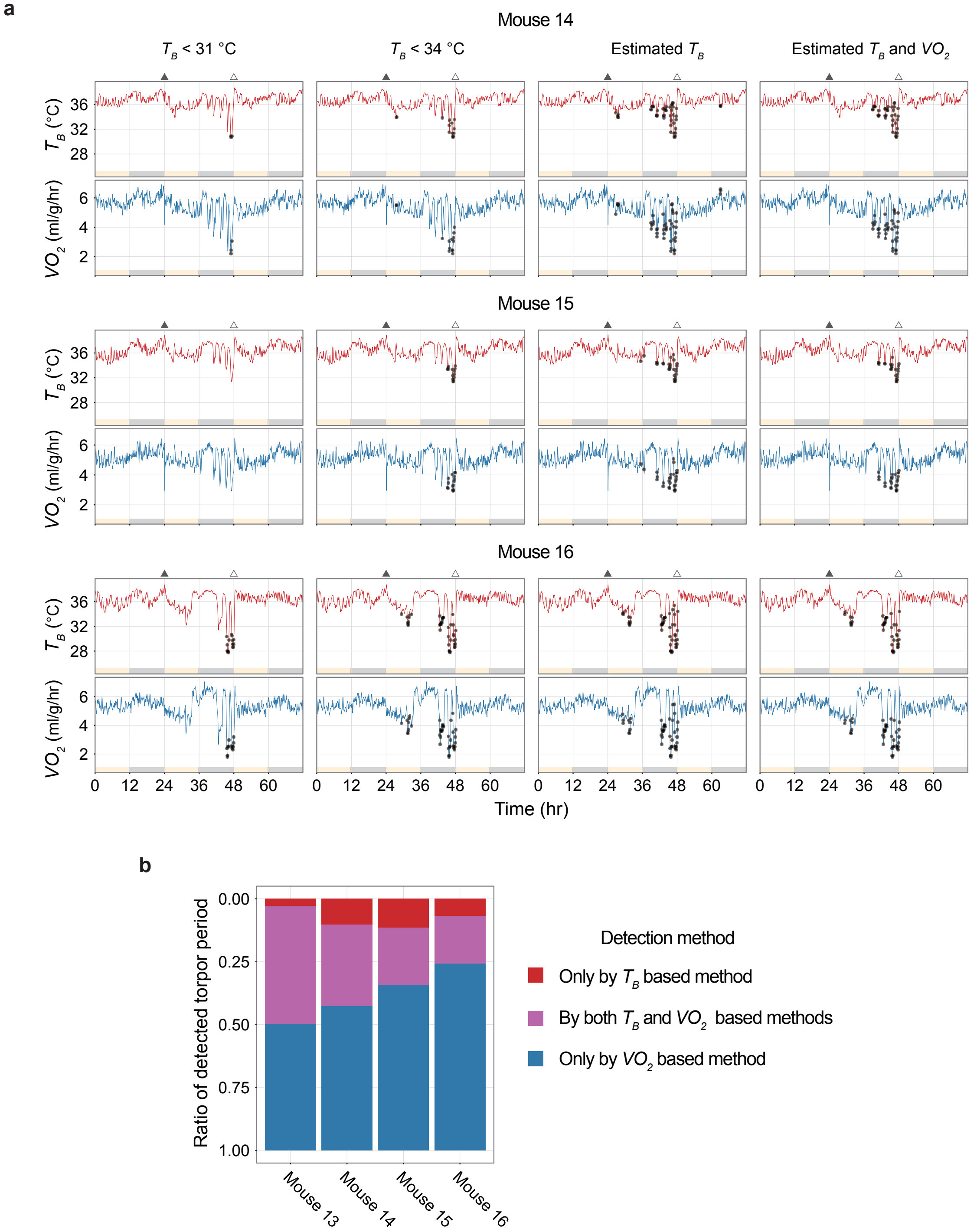

Sunagawa *et al.*, Supplementary Figure 3

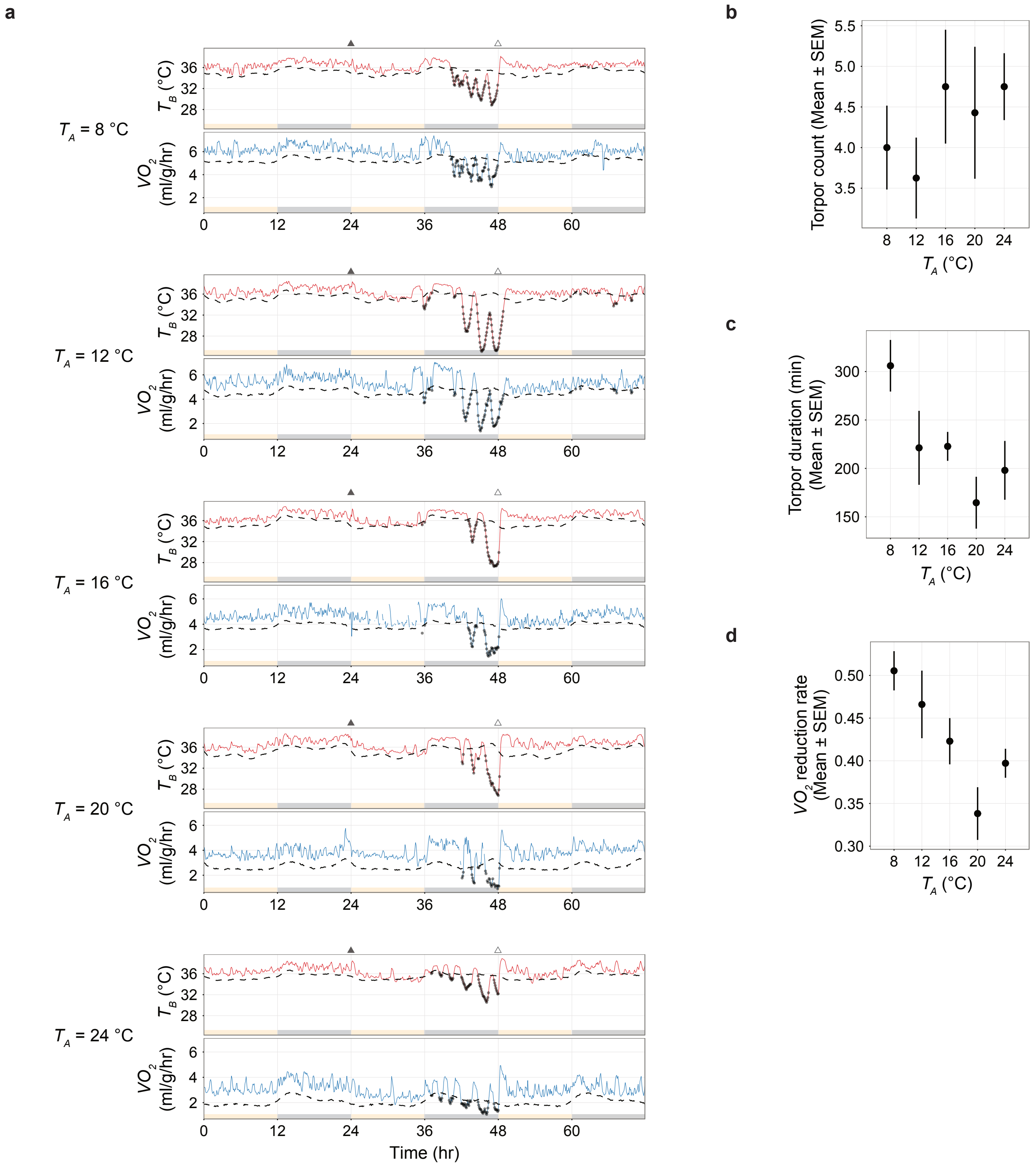

Sunagawa *et al.*, Supplementary Figure 4

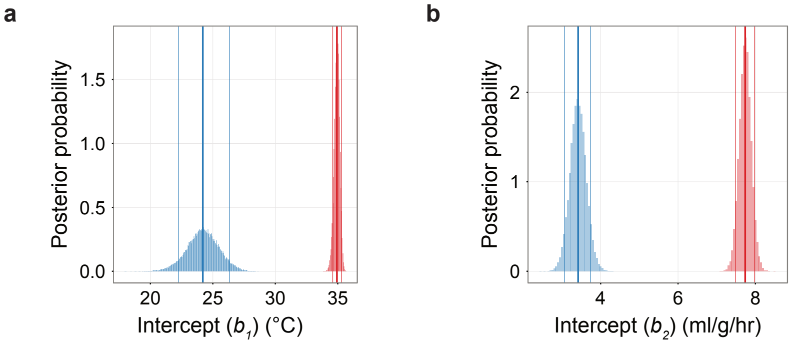

Sunagawa *et al.*, Supplementary Figure 5

```
// Supplementary Source Code 1
// Stan model for TB / VO2 modeling
//
// By Genshiro A Sunagawa (genshiro.sunagawa@riken.jp),
2016-07-12.
// This work is licensed under a Creative Commons
Attribution 4.0 International License.

data {
   int<lower=1> N; // time-point for one day (usually
240)
   real X[N]; // the observed value
   real sigma2;
}

parameters {
   real<lower=0> sigma1;
   real B[N]; // the base
}

model {

  B[1] ~ normal(2*B[N] - B[N-1], sigma2);
  B[2] ~ normal(2*B[1] - B[N],   sigma2);
  for (n in 3:N)
    B[n]~normal(2*B[n-1] - B[n-2], sigma2);

  for (n in 1:N)
    X[n]~normal(B[n], sigma1);

}

generated quantities{
  real X_new[N];
  for (n in 1:N)
    X_new[n]=normal_rng(B[n], sigma1);
}
```

```stan
// Supplementary Source Code 2
// Stan model for G, H and TR modeling
//
// By Genshiro A Sunagawa (genshiro.sunagawa@riken.jp),
2016-07-12.
// This work is licensed under a Creative Commons
Attribution 4.0 International License.


data {
   int<lower=1> N; // number of observations
   real TA[N]; // ambient temperature
   real TB[N]; // body temperature
   real VO2[N]; // energy output
}

parameters {
  real<lower=0> a1; // slope of TA-TB
  real<lower=0> a2; // - slope of TA-VO2
  real b1; // TB-intercept
  real b2; // VO2-intercept
  real<lower=0> sigma_VO2;
  real<lower=0> sigma_TB;
  real<lower=0> G;
}

model {

  a1~lognormal(0, 1);
  a2~lognormal(0, 1);
  G~lognormal(0, 1);

  for (i in 1:N){
    TB[i]~normal(a1*TA[i]+b1, sigma_TB);
    VO2[i]~normal(-a2*TA[i]+b2, sigma_VO2);
    VO2[i]~normal(G*(TB[i]-TA[i]), sigma_VO2);
  }

}

generated quantities{
  real H;
  real TR;
  real TA_TB[41]; // TA=0 to 40
  real TA_VO2[41]; // TA=0 to 40
  real TB_VO2[41]; // TB=0 to 40
  real TB_minus_TA_VO2[41]; // TB=0 to 40

  H=a2/a1;
  TR=(a2*b1+a1*b2)/a2;
```

```
  for (i in 1:41) TB_VO2[i]=(TR-(i-1))*H;

  for (i in 1:41) TA_TB[i]=normal_rng(a1*(i-1)+b1,
sigma_TB);
  for (i in 1:41) TA_VO2[i]=normal_rng(-a2*(i-1)+b2,
sigma_VO2);

  for (i in 1:41) TB_minus_TA_VO2[i]=normal_rng(G*(i-1),
sigma_VO2);

}
```
